# Coproducing an intervention to support stroke unit staff to provide information about recovery to patients and carers

**DOI:** 10.1016/j.pecinn.2026.100483

**Published:** 2026-06-12

**Authors:** Louisa-Jane Burton, Thomas Frederick Crocker, Oliver M. Todd, Sarah Bates, Anne Forster

**Affiliations:** a Academic Unit for Ageing & Stroke Research, Bradford Teaching Hospitals NHS Foundation Trust, Bradford, UK; b Academic Unit for Ageing & Stroke Research, Leeds Institute of Health Sciences, University of Leeds, UK; c Acute Stroke Unit, Leeds General Infirmary, Leeds Teaching Hospitals NHS Foundation Trust, Leeds, UK

**Keywords:** Coproduction, Stroke, Rehabilitation, Information provision, Communication, Patient and public involvement

## Abstract

**Objective:**

Stroke survivors and their carers report receiving insufficient information about recovery, particularly how much is expected and the associated timescales. Providing this information is challenging for stroke unit staff, due to concerns about maintaining patients' hope and motivation for rehabilitation, and a lack of training. We aimed to design an intervention to support staff to provide information about recovery in ways which meet patient and carer needs.

**Methods:**

Five stroke survivors, four carers, and six stroke unit professionals participated in six, monthly coproduction workshops.

**Results:**

The group created an intervention designed to increase staff confidence and skills in providing post-stroke recovery information. The intervention encourages a patient-centred approach and includes good practice guidance and a training package for staff (including communication skills training), with associated materials to deliver the approach.

**Conclusion:**

An evidence-based, theory-informed intervention has been coproduced, with the potential to meet the needs of intervention targets (staff) and information recipients (patients/carers).

**Innovation:**

A novel approach to designing an intervention to improve provision of post-stroke recovery information was employed. Inclusion of stroke survivor and carer perspectives aimed to ensure the intervention would meet patients and carers' needs, whilst professionals' perspectives ensured it was feasible to implement in practice.

## Highlights

• This is the first intervention designed to support stroke unit staff to provide information about recovery.
• The intervention was coproduced by stroke survivors, carers and stroke unit staff.
• The intervention encourages a patient-centred approach to information provision.
• Training for staff to improve their skills and confidence has been incorporated.

## 1 Introduction

### 1.1 Background

Stroke affects 100,000 people every year in the UK [1] and is a leading cause of disability worldwide [2]. Its effects can be sudden and lifechanging, impacting both physical and psychological functioning. Following acute treatment, survivors typically undergo a period of rehabilitation, receiving care from a skilled multidisciplinary team (MDT) of doctors, nurses, and therapists, who work with patients to improve their independence and quality of life. For many in the UK, rehabilitation is delivered in an in-patient stroke unit, from which ∼a third of patients are discharged requiring help with daily activities [1]. Whilst recovery can continue for months and even years after the stroke, it typically slows following the acute period, and many patients experience long-term disability [3]. This trajectory can be unexpected for patients and their families, who may feel unprepared for this eventuality when leaving hospital, and express dissatisfaction with information provided by stroke professionals about the likely timing and extent of their individual recovery, and the process through which this takes place [4], [5], [6]. Such information is required for them to participate in shared decision-making about their care, plan for the future, and facilitate adjustment to any continuing disability [7], [8].

UK guidelines direct professionals to compassionately convey individualised information about the likelihood that patients will meet their goals (usually recovery-related) [9]. However, professionals find providing information about recovery to patients and families challenging [10], [11], [12]. In our work, we use the term ‘information provision’ regarding recovery to mean communication between professionals and patients/carers, that includes at least one topic from: progress to date, recovery processes in general, and tailored prognostic information about the likely timing and extent of recovery [10]. This information is usually provided via two-way communication, primarily formal and informal conversations, with professionals, patients and/or carers as active participants. Although patients may initiate these conversations, they often do not; yet professionals make assessments about the likelihood and timing of future recovery to support clinical decisions about access to rehabilitation, its focus and timing of discharge [13], [14], [15].

Professionals find it particularly challenging to provide information about the likelihood and timing of future recovery where this involves the potential for ongoing disability [11], [12], [16]. They describe the need to balance conveying realistic information with motivating patients to continue their active engagement in rehabilitation and helping them maintain hope [11], [16], [17]. Predicting post-stroke recovery can also be difficult, although professionals develop skills and expertise through experience, which inform treatment plans and decision-making [10]; this clinical expertise is supported by the increasing development and availability of tools to predict functional outcomes [18]. Despite these developments however, professionals (particularly nurses and therapists) report little training in providing information about recovery to patients and families, resulting in a lack of confidence and potential avoidance, particularly around breaking bad news about potential long-term disability [11], [19]. This warrants the need for an intervention to improve provision of information about recovery by stroke unit staff to patients and their families.

Although such interventions have not previously been developed in the field of stroke, a systematic review of the effectiveness of interventions to improve provision of information about recovery in patients with neurological conditions [15] identified four studies [20], [21], [22], [23], [24], [25] testing their effects on patient outcomes. The identified interventions included an educational programme [22], personalised information provision [23], a decision-coaching intervention [20], [21] and a decision aid [24], [25]. Whilst none of the four interventions were specifically designed to improve provision of recovery information (incorporating this element as part of a wider programme), all included delivery of evidence-based information; tailoring of content to individual patients; and active patient engagement through discussion (versus passive information provision). Findings suggest these interventions, underpinned by the idea that information provision increases patients' sense of control in uncertain situations and empowers them in decision-making, could improve patient satisfaction with information and overall care [15].

Interventions to improve communication about recovery and prognosis have traditionally been developed using clinician-led and researcher-driven approaches. In the past decade, participatory methods including coproduction, co-design and co-creation (often known as the ‘co’-approaches and used interchangeably) have gained popularity in healthcare intervention development, to better align interventions with the needs of those they are intended to benefit [26]. These approaches draw on the knowledge and experiences of service users and providers and have been applied to developing interventions to improve the communication of prognostic information. For example, Hjelmfors et al. [27] used brainstorming, prioritising and prototyping with patients, their family members and healthcare professionals to co-design an intervention to improve communication around the heart failure trajectory. Similarly, Caven et al. [28] co-designed solutions to improve serious illness conversations (including sharing prognosis) with hospitalised patients, carers and healthcare providers; first exploring the problem and prioritising, then developing and refining solutions. These studies demonstrate that co-approaches are feasible and can produce interventions acceptable to patients, carers and healthcare professionals.

### 1.2 Aims

In this study, we aimed to use coproduction to design an intervention to improve the quality, consistency, and delivery of information about recovery by stroke unit staff to patients and their families.

### 1.3 Previous phases of the project

Our intervention development work has been informed by established guidance [29], [30]. Early systematic review work explored the experiences and views of those providing and receiving information about recovery in acquired neurological conditions, highlighting challenges experienced by professionals and their impact on patient experiences [11]. We further explored these challenges in a focused ethnographic case study in two English stroke units [10], [31], using non-participant observations (*N* = 84), interviews with staff (*n* = 19), stroke survivors (*n* = 10) and carers (*n* = 4) and documentary analysis of clinical records (*n* = 20). Analysis of these data underpinned early theoretically-based intervention development using the Behaviour Change Wheel (BCW). The BCW proposes that for an individual to exhibit a target behaviour, they must have the capability and opportunity to do so, and be more motivated to exhibit this behaviour than any alternative [32], [33]. In our work, the target behaviour was “stroke unit professionals provide information about recovery to patients with stroke and their carers” [34]. Preliminary work identified twelve barriers linked to capability, opportunity, and motivation, six potential Intervention Functions (strategies linked to capability, opportunity and motivation that could theoretically be effective in changing behaviour), and 29 relevant Behaviour Change Techniques (BCTs; active ingredients of an intervention) [34]. Subsequent stakeholder involvement collected views via an online survey from 48 stroke unit professionals on the importance of addressing these barriers and potential feasibility and usefulness of the selected BCTs [34]. This work supported the transferability of earlier findings to other stroke units and informed decision-making.

## 2 Methods

We report our intervention development consistent with the GUIDED reporting guideline [35] (see Supplementary File 2).

### 2.1 Study design

Grounded in a pragmatic paradigm, we selected methods aligned with our research aims. We adopted a coproduction approach to ensure the designed intervention was contextually relevant and shaped by the lived experiences of intended users. Although a single definition of ‘coproduction’ does not exist, our approach aligns most closely with Smith et al.'s third typology of ‘Equitable and experientially-informed research’ [36]. Here, participants' lived experiences are central, and equitable partnerships (in this case, between patients, carers and professionals) are developed, with an emphasis on power-sharing [36]. We were also guided by the NIHR's key principles, including sharing power; including all perspectives and skills; respecting and valuing all knowledge and contributions; reciprocity; and building and maintaining relationships [37]. Although our intervention aimed to change staff behaviour (and service users may know less about professionals' practice and motivations), we felt it important to work collaboratively with stroke survivors and carers to ensure that the intended practice changes would meet their needs.

**Fig. 1:**
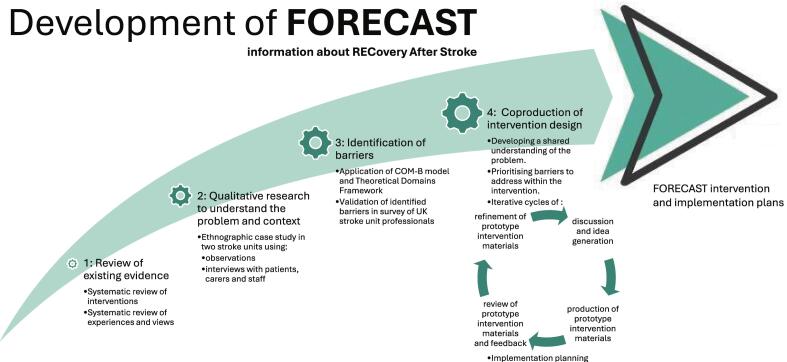
Phases of development of the FORECAST (Information about recovery after stroke) intervention.

Intervention design was informed by O'Cathain's guidance [30]: generating ideas about content, format and delivery with stakeholders, developing a prototype and iteratively refining it based on its acceptability, feasibility and how engaging it is (see Fig. 1).

### 2.2 Study concepts

We draw on Bernhardt et al.'s conceptual framework of stroke recovery [38], which delineates between recovery as a change in outcomes over time and the mechanism through which this occurs (restitution of normal pre-stroke function or the modification of the patient's behaviour to accomplish tasks within the constraints of their residual impairments (compensation)) [38]. In this study, we primarily apply the former representation, which may include changes in body functions and structures, activities and participation in society [38], [39], whilst recognising that conversations about recovery may also include *how* such changes occur.

Whilst recovery from stroke is rarely complete, improvement may continue for months or years [3]. Although many stroke survivors initially expect a return to pre-stroke function [40]; most experience lasting effects, and a period of adjustment is required as they work to make sense of their new reality [41]. In this paper, we use ‘adjustment’ to refer to the ongoing process of adapting to and accommodating these changes over time [42].

Throughout their ongoing recovery, many stroke survivors are supported by informal carers; in this study we use this term according to Luker et al.'s definition: *“the spouse or partner, family members, friends or ‘significant others’ who provide physical, practical or emotional support to someone after their stroke”* pp. 1852–3 [43]*.*

### 2.3 Ethics

Ethical approval was received from the North West – Greater Manchester West Research Ethics Committee (REC reference: 23/NW/0243). All participants provided written informed consent.

### 2.4 Study setting

This study was conducted in the UK, where clinical guidelines [9] mandate that stroke care and rehabilitation is provided in dedicated hospital-based stroke units, where MDTs with specialist stroke expertise deliver coordinated care supported by education and training programmes [44]. Core professionals include physiotherapists, occupational therapists and speech and language therapists, nurses and doctors [9], supported by therapy assistants and support workers. Whilst therapy teams typically comprise some senior staff, most day-to-day rehabilitation is provided by those with less experience, who may regularly rotate around different specialties.

Despite guidelines recommending discussion of recovery with patients and families, there is limited guidance for professionals about who should provide this information and how it should be delivered. As a result, practice varies: In some units, information is shared in multidisciplinary family meetings, where staff collectively provide this information to patients and families; in others, information is delivered by individual therapists [10]. Previous research has highlighted how nurses' and therapists' skills in predicting and communicating about recovery is primarily learned through experience, with limited or no formal training, which can be challenging for junior rotational staff [31], despite regular clinical supervision. Doctors typically receive communication skills training within medical education, although this frequently focusses on life-limiting conditions such as cancer.

Pragmatically, the study was conducted near to the research team in northern England, UK. Stroke survivor and carer participants were recruited from the surrounding area, to facilitate convenient travel and minimise logistical barriers to participation, particularly for people who may experience fatigue. By recruiting from this locality, we aimed to reduce participant burden and enhance attendance and retention throughout the study; stroke survivors/carers were not required to have necessarily accessed stroke services within this area.

Staff were recruited from two stroke units within one NHS Trust. One unit provided hyper-acute care, acute care and early rehabilitation for patients with stroke; a relatively short length of stay meant information about recovery was primarily delivered by individual staff members. The second unit provided longer-term rehabilitation, with information delivery about recovery supported by regular family meetings and key workers who acted as intermediaries with the clinical team. Neither stroke unit provided formal training about discussing recovery.

### 2.5 Participants

Participants were required to be English-speaking (due to resource limitations) and able to provide informed consent to participate (to meaningfully contribute to discussions). Inclusion criteria for each participant group were:

*Stroke survivors:* Aged ≥16 years; self-reported diagnosis of stroke; received in-patient rehabilitation in a stroke unit; living in the community.

*Carers:* Aged ≥16 years; family member/close friend of someone who received a diagnosis of stroke and received in-patient rehabilitation in a stroke unit; living in the community.

*Staff:* Aged ≥16 years; current member of a stroke unit MDT with a professional qualification.

We aimed to sample 12–15 participants; our intention was to include people with a range of diverse experiences whilst maintaining a small enough group to facilitate relationship-building and creating space for all to express their views, aligned with guidance [37]. This also allowed for potential drop-out.

We adopted a purposive approach to sampling, identifying participants with a range of experiences to contribute. Stroke survivors were selected to include those with a range of post-stroke severities and effects, including cognitive and communication difficulties. Stroke survivor and carer participants were recruited from two nearby community-based stroke groups. A researcher visited the groups to provide study information; those interested completed a ‘consent-to-contact’ form. Eligibility was assessed via telephone; a subsequent home visit included detailed discussion about the study, informal capacity assessment, and informed consent.

For professionals, we aimed to sample participants from each of the core stroke unit disciplines (medical, nursing, physiotherapy, occupational therapy, speech and language therapy, psychology). Dissemination of study information was supported by SB (a senior physiotherapist from the Trust); those interested contacted the lead researcher (LB) if they wished to take part.

### 2.6 Data collection and analysis

#### 2.6.1 Demographic data

For stroke survivors, demographic data including age, gender, time since stroke, presence/absence of aphasia, pre- and post-stroke occupational status, and degree of disability (self-reported modified Rankin Scale [45]) were collected at recruitment. For carers, we collected age, gender, relationship to the stroke survivor and change in caring responsibilities following the stroke. For staff, we collected age, gender, profession, seniority, time since qualification and time working in stroke services.

#### 2.6.2 Coproduction of intervention design

Plans for the overall structure of the coproduction workshops were determined in advance, progressing from introduction of the topic and prioritisation, through cycles of idea generation, prototyping and feedback; the content however was determined by the group. At the start of the process, aligned with guidance [37], participants agreed ‘ground rules’ for contributing, including confidentiality and respect. Early discussions focused on introducing the complexity of the topic in accessible ways and achieving participant buy-in. This began with an introduction to coproduction, and used vignettes to elicit discussion about important factors for patients, carers and professionals regarding providing and receiving information about recovery in a stroke unit. The aim of this initial exercise was to support understanding of different perspectives around potential challenges.

##### 2.6.2.1 Prioritising barriers to address within the intervention

Participants then took part in a card-sorting exercise, designed to help them prioritise which barriers to providing information identified from our previous work [34] were most important to address. This exercise facilitated participant engagement and ownership over the intervention development process. Priorities were agreed by the whole group.

##### 2.6.2.2 Generating ideas, developing prototypes and iterative refinement

Work proceeded with generating ideas collaboratively, then prototyping solutions (see 2.7). Content (including worksheets and materials) and solutions were developed iteratively during the process, according to participants' identified priorities and refined in accordance with their feedback.

##### 2.6.2.3 Implementation planning

Implementation was considered throughout, with specific discussions around the timing of information provision, and which staff should be involved. Ultimate decisions around implementation were made in the final stages. In line with O’Cathain's guidance, which recommends articulating programme theory, we developed a logic model using the structure developed by Smith et al. [46].

#### 2.6.3 Workshop delivery

To facilitate the collaborative group interaction underpinning the coproduction, six face-to-face workshops were organised. Workshops typically encompassed a short researcher-led presentation and two forty-five-minute group-based activities. Workshop activities (detailed in Table 1) were developed responsively based on the decisions of the coproduction group, e.g., the existing models reviewed in Workshop 2 reflected the priorities identified in Workshop 1. Efforts were made to develop materials and run workshops in ways that supported participant engagement. This included ensuring worksheets and activities were accessible (particularly for those with communication and cognitive difficulties) and easy to understand (supported by a stroke survivor member of our 10.13039/100006163Project Management Group). Materials enabled participants to express their views pictorially or in writing, as well as verbally. Where needed, research team members supported individuals to express their views.

**Table 1:** Overview of workshop (WS) activities.

Workshop	Objective	Methods
WS1	To introduce coproduction method and generate shared understanding of the challenges to providing and receiving information about recovery from different perspectives. To prioritise barriers to address within the intervention.	Someone Who Isn't Me (SWIM) technique [47]: Based on findings from previous ethnographic work, summaries were created for a patient with stroke, his carer and treating professional. In small groups, participants reviewed one summary and discussed the views and challenges experienced by the character, leading them to consider what a ‘good’ conversation about recovery looks like. Findings were shared with the wider group. Card-sorting: participants sorted the barriers identified in our previous work into piles according to how important they felt they were to address in the intervention (very important/quite important/not important). In small groups, participants discussed each barrier and ranked them from most to least important to address.
WS2	To agree priorities for the intervention. To identify learning and transferability from existing models of ‘breaking bad news’ in other conditions and consider how challenges specific to stroke could be addressed.	Presentation of results from WS1 card-sorting task, and comparison with results from previously conducted staff survey. Whole group discussion to agree priorities for the intervention. Small group discussions: Each group reviewed one model of ‘breaking bad news’ in healthcare settings, e.g., the Setting, Perception, Invitation, Knowledge, Emotions, Strategy/Summary (SPIKES) protocol [48], identified in our earlier review work [11]. Participants considered whether the elements of the model were relevant to/important in stroke care and fed back to the wider group what worked and what would require changing. Presentation of challenges specific to stroke identified from previous ethnographic work/existing literature. Group discussions around how these challenges could be addressed.
WS3	To review a prototype of ‘good practice’ guidance for communication of information about recovery for use in stroke care, developed from analysis of discussions from previous workshops. To identify what we would measure to understand if our intervention had been effective. To identify the roles of different staff groups in delivering information about recovery.	Small group discussions: Review of sections of guidance document. Groups worked together to refine the guidance (including content and wording). Professionals were asked to consider whether the guidance was feasible to deliver, too restrictive/flexible. Stroke survivors and carers were asked to consider how they would have felt if professionals had delivered information according to the guidance when they had their stroke. Presentation of potential measures that could be used to assess whether the intervention was effective in a future feasibility study, with small group discussion (stroke survivor/carer participants only). Small group discussion (professional participants only) around the roles of different staff groups and of different experience levels in the process of providing information about recovery to stroke survivors and families.
WS4	To begin development of supporting materials for professionals to provide to patients when discussing the stroke recovery process. To review a prototype tool to support staff to identify patients/carers' needs and preferences for information about recovery.	Patient pathway mapping: In small groups, participants worked together to map the patient pathway through stroke services and beyond. Participants used post-it notes to record questions they had/ information they could provide at each stage. Small group discussions: Participants reviewed and refined the tool, considering content, wording, whether anything was missing, how they would feel about asking/answering the questions. Participants discussed how the tool could be used in practice, including how it should be completed, who should complete it, when and how often, and how information gathered could be shared across the multidisciplinary team.
WS5	To review prototype intervention components developed in WS4 (patient journey map, communication starter tool). To decide how these components should be implemented.	Small group discussions: Participants reviewed prototype intervention materials and commented on their content and format. Small group discussions: Participants discussed how the materials could be used in practice (who provides, to whom, when, how the materials are introduced). Professional participants discussed the feasibility of plans; stroke survivor/carer participants commented on acceptability and described their preferences.
WS6	To review intervention components and consider how the intervention will be implemented in practice. To reflect on and evaluate the coproduction process.	Small group discussions: Participants reviewed intervention components and discussed how they could be implemented and used in practice. Individual questionnaires (with versions for stroke survivors/carers and staff) were completed, with questions around engagement and what went well/less well. Small group discussions provided an opportunity for participants to jointly reflect on their experiences and the impact their participation had.

Workshop dates were pre-arranged prior to the start of the project and held face-to-face in an accessible location at the university. Although we had planned to involve participants in decisions around timings, potential participants preferred to know the dates and times in advance to inform decisions about participation. The venue was selected (following conversations with potential participants) due to its proximity to a hospital where some staff participants were based, and the city centre, facilitating access for stroke survivors and carers. Each session lasted three hours (11 am-2 pm), with lunch and regular breaks provided. Stroke survivor and carer participants were compensated for their time (using vouchers) and the staff members' employing NHS Trust was recompensed for staff time. Participant travel expenses were reimbursed.

Workshops were led by LB and facilitated by two/three stroke researchers. Group-based activities were conducted in three smaller groups of three to five participants, each with mixed representation from each participant group and a facilitator. Facilitators were briefed prior to each session; their role was to facilitate discussions (encouraging quieter voices, supporting participation, maintaining focus), and contribute relevant research evidence where appropriate. Small group composition varied across meetings, to enable participants to work closely with all members. In a debriefing session following each workshop, facilitators reflected on participant engagement in each task and how the groups had worked together to accomplish it; alongside participant feedback, this informed development of subsequent tasks and group allocation. With participants' consent, discussions were audio-recorded, with some transcribed, to inform development of intervention materials.

Following each workshop, participants completed an evaluation questionnaire, to assess their engagement with and understanding of the coproduction process and gather feedback to improve experiences at subsequent meetings. At the final workshop, participants completed a longer evaluation questionnaire, reflecting on their experiences, engagement, perceived benefits of and challenges to the process, and evaluation of the developed intervention (data to be published separately).

Data collected throughout the process included completed worksheets, audio recordings and transcripts of small group discussions, and evaluation questionnaires.

### 2.7 Data analysis

Demographic data were summarised descriptively. LB reviewed audio recordings and worksheets from group discussions to draw out key insights to support the generation and refinement of prototype intervention materials. These summaries were discussed with the facilitators and research team. As the aim was to summarise the group's ideas, formal qualitative analysis methods were not employed. However, we drew upon established qualitative techniques to ensure a systematic approach to summarising participants' ideas. For example, to generate draft recommendations for the ‘good practice guide’ (see 3.3.3), we drew upon conventional content analysis [49]. LB reviewed and transcribed the group discussions from Workshops 1 and 2, familiarising herself with the content before open-coding the transcripts line-by-line. Similar ideas were grouped into categories and combined into recommendations within each category. These were iteratively reviewed and refined through dialogue with the research team and coproduction group, ensuring validation and shared decision-making. The recommendations were subsequently presented in a purposely designed booklet to the coproduction group for further review and feedback.

Other intervention components were developed by reviewing audio-recorded discussions and written materials, summarising ideas, and refining them collaboratively. For example, patient journey maps (see 3.3.5) from Workshop 4 were consolidated into a single map, reviewed and refined by the group, and implementation discussed in Workshop 5. One component was developed by a sub-group comprising the professional participants alongside LB because of the need for professional insight to contribute (staff role descriptions; see 3.3.3). Another component, staff training, was developed in partnership with experienced palliative care nurses skilled in delivering similar communication skills courses, and summaries were discussed with the coproduction group for refinement and approval.

Throughout their development, intervention components were linked to Behaviour Change Techniques (and related to those previously identified as having potential to be effective in our earlier work [34]).

Engagement with and success of the co-production process were assessed through collection of interactive materials completed by workshop participants and responses to the evaluation surveys (reported separately).

### 2.8 Trustworthiness

We employed strategies to ensure trustworthiness in our research [50]. Credibility was enhanced through triangulation of perspectives (stroke survivors, carers, staff), and sources (written notes, completed worksheets and audio-recordings of discussions), and active exploration of divergent views. Dependability was supported by an audit trail documenting recruitment, data collection and group decisions made during intervention design. Confirmability was strengthened through reflexivity (detailed below) and collaborative validation with the coproduction group, ensuring transparency and minimising bias. Transferability was addressed through detailed description of the context in which our work took place, enabling application in similar settings.

### 2.9 Reflexivity

The lead researcher (LB), a postdoctoral researcher whose doctoral studies in this topic were completed under the supervision of two co-authors (AF and TC), led the workshops and continually reflected on how her prior research experience and knowledge of the literature might shape her interpretations and questioning. Participants were introduced to LB and all workshop facilitators along with their professional roles. Whilst most were researchers with no clinical background, three facilitators had a background in physiotherapy and stroke care. Facilitators were also asked to reflect on how their previous experiences could have influenced small group discussions; these reflections were documented and considered by LB when reviewing audio-recordings. Insights from this process informed adjustments for subsequent workshops. We also acknowledged that LB's expertise and facilitators' roles could impact power dynamics within the sessions; to mitigate this, participants were regularly reassured that their knowledge and experience were equally valuable and offered perspectives that the research team could not access without their input.

## 3 Results

### 3.1 Participants

Five stroke survivors, four carers, and six professionals participated. Stroke survivor participants' ages ranged from 35 to 84 years (mean age = 63 years). Three were female (60%); all were white British. Time post-stroke was between 18 months and 6 years (mean = 46.6 months). Modified Rankin Scale score ranged from 2 (slight disability) to 4 (moderately severe disability). Two reported aphasia. All recruited carers were a spouse of a participating stroke survivor. They ranged in age from 43 to 86 years (mean age = 70 years). Half were female; all were white British. All reported their caring responsibilities increased following the stroke; three reported being full-time carers, one was a part-time carer.

The six recruited professionals included a physician associate, nurse, physiotherapist, occupational therapist, speech and language therapist and clinical neuropsychologist. Four were female (66.67%); all were white British. Participants ranged in age from 29 to 40 years (mean = 33.5 years), and in their experience in stroke care (<1 year *n* = 1; 1–5 years *n* = 2, 6–10 years n = 1; >10 years n = 2).

### 3.2 Workshop attendance

**Fig. 2:**
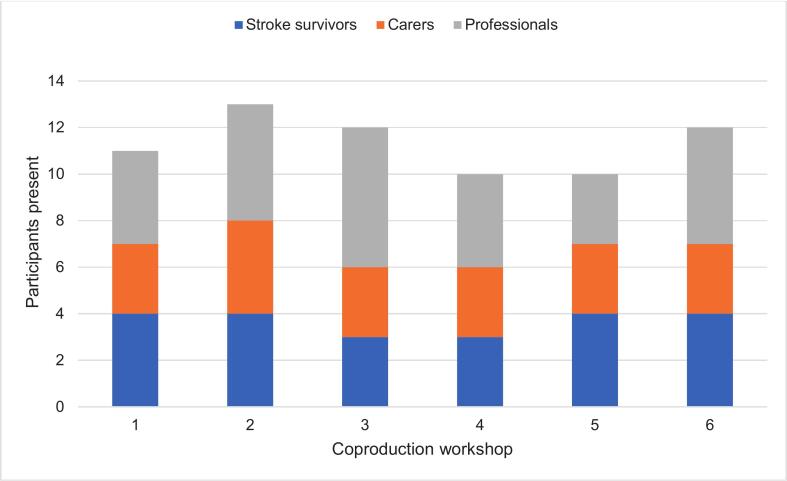
Workshop attendance. Bar chart displaying the number of participants present at each workshop.

Workshops were well-attended, with ten to 13 (67–87%) of the 15 recruited participants present at each (see Fig. 2). One stroke survivor attended only the first meeting. Non-attendance was a result of clinical pressures, or annual leave (staff), illness, or life events (e.g., funeral attendance).

### 3.3 The FORECAST intervention

Briefly, the final prototype intervention aims to improve stroke unit processes and multidisciplinary staff skills and confidence to deliver a proactive, regular approach to provision of information about recovery (primarily through verbal discussions, with supporting written information where needed), in ways which are clear, compassionate, and tailored to patients' and carers' individual needs and preferences. The intended patient-centred approach to information provision involves staff implementing routine processes to:

• Identify individual patients' preferences and needs for information about recovery (supported by a structured checklist: the ‘conversation starter tool’);
• Provide a map of a typical recovery journey, highlighting how services support recovery (the ‘patient journey map’);
• Regularly and proactively discuss recovery with patients and carers, including providing verbal information tailored to meet their needs (e.g., supported by written notes if desired/required).

The intervention includes good practice guidance and a training package for staff (including communication skills training), with associated materials to deliver the approach. Training is delivered across two sessions: session 1 is for all staff, introduces the approach and materials, and provides training in active listening; session 2 is specifically aimed at those involved in delivering recovery predictions (i.e., senior members of staff), providing training in communication skills and strategies.

**Fig. 3:**
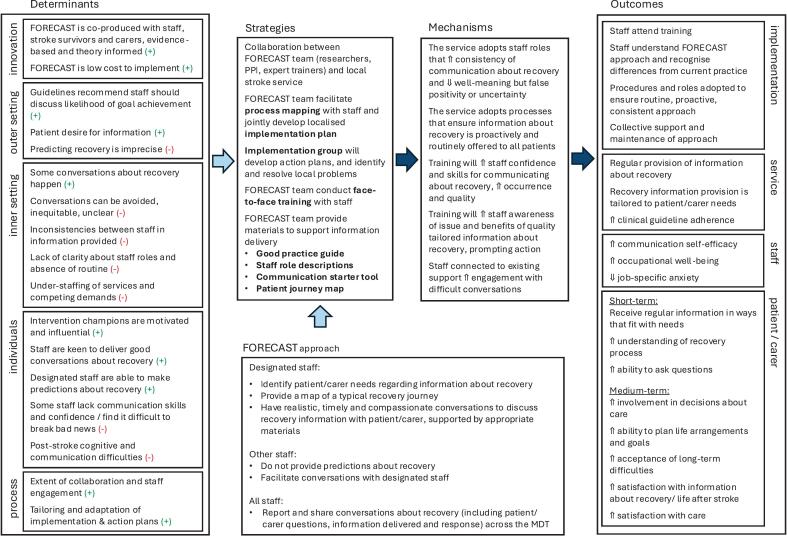
Logic model of the FORECAST intervention showing the target approach, barriers (-) and facilitators (+) that inform the strategies, the intended mechanisms of action and anticipated outcomes. ⇑ = increase; ⇓ = decrease.

Fig. 3 presents the logic model of the FORECAST intervention. Key insights from participants and how they were incorporated into the final intervention are detailed in Table 2. The following sections provide details of the intervention design and content, including how workshop participants contributed.

**Table 2:** Key insights emerging from participants' discussions and how they were incorporated into intervention design and content.

Theme	Key insights	How incorporated into intervention
Individual needs and preferences	Participants highlighted that some patients/ carers may not wish to know about their likelihood of achieving their recovery goals (and that the wishes of patients and carers may not be congruent). They also discussed how some patients may not be ‘ready’ to hear information about their likely progress early in their admission, due to stroke-related difficulties (e.g., decreased awareness, cognitive problems, difficulties in understanding) and feeling overwhelmed.	Recognition of individual preferences for receiving recovery information and needs led to recommendations for a patient-centred approach to information provision (i.e., not at specific time-points such as two weeks post-admission). The **good practice guide** recommends engaging in dialogue with patients and carers to understand their information needs and how they can best be met. The **communication starter tool** was developed to address these concerns and is designed to be used to open the dialogue between staff and patients/carers about the types of information they wish to know, and how they would like such information to be provided (e.g., verbally, in writing, with a single staff member, in a family meeting). There are opportunities to practice its use in the **training package** (Session 1).
Definition of ‘recovery’ and expectations	Participants discussed that patient and carer expectations for recovery related to a ‘return to normal’ including performing everyday activities in the same way they did pre-stroke. Professional participants highlighted their views that this was unlikely for many, and that they sought to maximise (through therapy) patients' function, activity levels and quality of life, anticipating some level of residual disability for most.	The **training package** includes videos of stroke survivors discussing their recovery expectations (highlighting how patients' expectations may differ from those of staff). It also provides guidance to staff around assessing patients and carers' expectations, e.g., through ascertaining prior knowledge of stroke and the source of such knowledge. These issues are also highlighted in the **good practice guide**.
Importance of hope	Stroke survivor and carer participants stressed the importance of maintaining hope for their recovery; for a minority, this meant not receiving predictions about their potential for recovery, however most felt that the best approach was for staff to provide honest information, whilst allowing them to maintain hope for their life after stroke.	Highlighting the importance of hope whilst providing realistic information forms a core part of the intervention and is discussed in the **training package** and the **good practice guide**. This includes practical suggestions for staff, e.g., providing information about services available to help patients/ carers (supported by the **patient journey map**), and highlighting different ways to accomplish activities.
The need for kindness and compassion when providing recovery information	Some stroke survivor/carer participants described positive experiences of receiving recovery information, including ‘bad news’. They discussed how these experiences were facilitated by staff kindness and compassion. Some believed that some people were naturally better communicators than others; however, all felt that communication skills could be taught, and that this would result in improvements to patient care. Professional participants described training in communicating about recovery and particularly in breaking bad news as an unmet need, which was particularly important as engaging in these conversations was a key part of their role.	The **training package**, which includes communication skills training is a key part of the intervention. It has been developed in collaboration with specialist palliative care nurses with significant experience of creating and delivering such training and is designed to be delivered by skilled trainers. Session 1 (all staff) includes active listening skills, which are important for staff to understand patients' and carers' concerns relating to their recovery. Session 2 (staff involved in delivering recovery predictions) including strategies for breaking bad news, with opportunities for role play, and feedback.
An absence of proactivity	There was a sense from stroke survivor/carer participants that professionals were not proactive in discussing post-stroke recovery. They described their experiences of trying to seek out the appropriate professional from whom to receive information, which could feel frustrating, and worried for those who did not have family to advocate for this information for them.	Within the **good practice guide** and **training package**, staff are encouraged to routinely begin a dialogue with all patients around their needs and wants for information about recovery (supported by the **conversation starter tool**) and subsequently provide information to meet these needs.
The roles of different staff members	Stroke survivor/carer participants generally described a preference to receive information about their likelihood of recovery from a doctor, though some reflected that the most useful information they had received had been from therapists. Professional participants highlighted the need for a shared understanding across the multidisciplinary team about whose role it was to discuss recovery (and where questions should be referred to colleagues). An identified need for patient/carer education about the expertise of different multidisciplinary team members was highlighted.	**Role descriptions** for team members form a core part of the intervention, enabling staff to understand their role in information provision (including how to respond to questions). Within the **training package** (session 1), the roles of different team members are discussed, alongside how patients and carers can be educated about the specialist roles of the MDT.
Understanding ‘what comes next’	Stroke survivor/carer participants discussed how, in the acute phase of stroke, they were unaware of how long they would remain in hospital, and that they were likely to have ongoing difficulties upon discharge. They described a lack of awareness about how services were organised to support their continuing recovery after leaving hospital, which could cause anxiety.	The **patient journey map** was developed to provide staff with a tangible piece of written information (which patients and carers could later refer to), which highlighted the post-stroke recovery journey and how services were organised to support it.
Unpredictability of post-stroke recovery	The unpredictability of post-stroke recovery was viewed as a challenge to information provision. Stroke survivor/carer participants described wanting information despite the potential for uncertainty, as long as this was expressed. Professional participants described potential for discomfort when conveying uncertainty. All participants highlighted the importance of having regular conversations that could reflect changes, particularly as the patient progressed. Professional participants highlighted the potential for (particularly junior) staff to respond to questions about recovery with unhelpful answers that suggested that no predictions can be made (e.g., *‘how long is a piece of string?’* or *‘if I had a pound for every time someone asked me that!’*), which could be unhelpful when later discussing predictions, e.g., in relation to discharge and support required.	Both the **training package** and **good practice guide** include training and advice for staff in conveying uncertainty (e.g., highlighting that any predictions are not ‘set in stone’ and the importance of providing uncertain information (if desired). The **conversation starter tool** is recommended to be used at regular intervals, so that changes in patients' desire for information can be captured and their needs met. The **training package** (Session 1) includes a section on *‘what not to say’* when faced with questions about recovery, and how these questions should instead be answered (e.g., explaining current uncertainty, arranging to revisit the subject at a set date or referring to a more experienced colleague).

#### 3.3.1 Intervention priorities

**Fig. 4:**
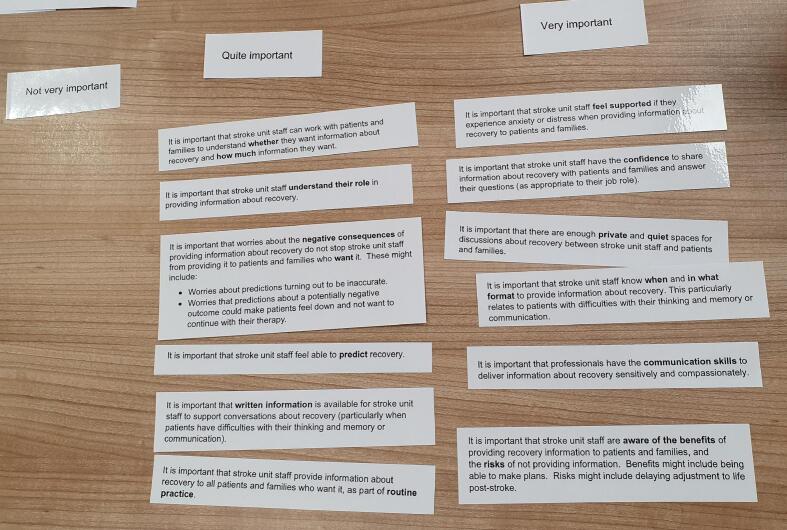
Example card sort of barriers to information provision. Photograph of cards each representing a barrier to provision of information about recovery, as sorted by one participant.

Improving staff skills and confidence in providing information were identified as key priorities by the group (Fig. 4). This correlated with findings from our previous stakeholder engagement work [34].

#### 3.3.2 Learning and transferability from existing models of ‘breaking bad news’

Given the identified priorities, the group considered whether and how existing models and tools to support communication of ‘bad news’ in healthcare could be introduced or adapted for stroke units. The models promoted significant discussion, with agreement on many key components; however, the group ultimately decided that a simple model would not be useful in this context, for two reasons. Firstly, the multidisciplinary nature of stroke unit care requires the coordination of information provision across a range of professionals with specialist expertise, whereas existing models are based on a single consultation between one staff member and a patient. Secondly, the group felt it important for all staff (both senior and junior) to understand their roles in discussing recovery with patients and carers, particularly when faced with unexpected questions outside formal meetings (e.g., ‘corridor conversations’), which existing models do not accommodate.

#### 3.3.3 Good practice guide, staff role descriptions and staff training package

Based on learning from the prioritisation work and review of existing models of ‘breaking bad news’, a ‘good practice guide’ covering issues specific to stroke units was developed. These issues included the unpredictability of post-stroke recovery and the challenges of maintaining patient hope and engagement in active rehabilitation whilst providing realistic information about likely outcomes. The guide is intended to be used by all stroke unit staff, regardless of seniority or profession, to support understanding and application of the FORECAST approach. ‘Role descriptions’ for junior, mid-grade, and senior staff were also developed to specify the skills required at each level.

Formal communication skills training was identified by the group as an essential component of the intervention. This was developed using evidence-based techniques (from research literature and expert guidance), including demonstration, role play and feedback and refined using learning from the workshops to ensure relevance to the stroke unit context. The training included approaches such as active listening and strategies for breaking bad news and was incorporated into a wider package detailing the intervention materials and guidance for their use. The package also included video-recorded interviews with stroke survivors and carers from the group, developed as a powerful way to help staff understand their perspectives, particularly related to the impact of receiving (or not receiving) information about recovery, and how it is delivered.

#### 3.3.4 Conversation starter tool

The group highlighted that stroke survivors differed in the amount, type and mode of information they preferred (e.g., verbal, written, in the presence of family). Although national guidelines recommend an individualised approach to information delivery [9], several professionals reported not routinely discussing these preferences, whilst; stroke survivors and carers described a desire to be asked. As a result, a ‘conversation starter tool’ was developed to help staff identify patients' needs and preferences. It was agreed that this tool could be provided to patients/carers, completed alongside them or be used by staff to prompt discussion, depending on clinical judgement.

#### 3.3.5 Patient journey map

Stroke survivor and carer participants described uncertainty about what to expect during recovery, including availability of services and how these fit together; professionals also desired some written information to support conversations about the stroke pathway. Participants collaboratively mapped the patient journey, with professionals contributing information on service organisation and stroke survivors and carers identifying the information they found helpful at each stage, and other forms of support accessed outside the NHS. This formed the ‘patient journey map’.

Mapping of intervention components to the BCW is displayed in Table 3. The final intervention is described using the Template for Intervention Description and Replication (TIDieR) framework [51] (see Supplementary File 1).

**Table 3:** Intervention components, mapped to the Behaviour Change Wheel [33].

COM-B domain requiring change	Barrier	Intervention function	BCT		Intervention component(s)
Psychological capability	Some stroke professionals perceive that they do not have the required communication skills to deliver information about recovery, particularly where this involves breaking bad news	Training	Instruction on how to perform a behaviour	Be provided with instructions about how to discuss recovery sensitively and compassionately	Good practice guide Staff training package
			Behavioural practice/ rehearsal	Practice conversations about recovery through role play with peers	Staff training package
			Feedback on behaviour	Receive feedback following observation of practice conversations with peers	Staff training package
	Some professionals find it difficult to assess **whether** and **how much** information patients and families want to know about recovery Some professionals find it difficult to decide **when** and **in what format** to provide information about recovery to meet individual patients' needs, e.g., where patients have cognitive or communication problems.	Training	Instruction on how to perform a behaviour	Receive advice on how to ask patients and carers about whether and how much information about recovery they wish to receive, and in what format	Good practice guide Staff training package Conversation starter tool
			Behavioural practice/ rehearsal	Practice asking whether and how much information is wanted and how best to provide it through role play with peers	Staff training package
			Feedback on behaviour	Receive feedback following observation of practice conversations with peers	Staff training package
Physical opportunity	Little written information about recovery is available for professionals to support conversations, particularly for patients/ families with cognitive or communication problems	Environmental restructuring	Adding objects to the environment	Be provided with generic written information to give to patients/families	Patient journey map
Reflective motivation	Some professionals are unaware of the benefits of providing recovery information to patients and families (e.g., making future plans or adjusting to life post-stroke), and the risks of not providing information (e.g., limiting ability to plan, preventing adjustment)	Education	Information about social and environmental consequences	Be provided with information on patients' and carers' information needs about recovery from established literature Be provided with information about the benefits of providing information about recovery to patients and families, e.g., to support adjustment, enable planning, but steps should be taken to ascertain how much/ the type of information they want to receive and to convey uncertainty	Good practice guide Staff training package
			Information about emotional consequences	Be provided with information about the emotional consequences for patients and carers if information about recovery is not provided effectively	Good practice guide Staff training package
		Persuasion	Credible source	View a speech by an expert (stroke survivor or carer) outlining the known benefits and risks to providing information about recovery	Staff training package
	Some professionals (particularly junior staff) report a lack of confidence in sharing information about recovery with patients and families, which may lead them to avoid providing information or providing vague information	Enablement	Social support (unspecified)	Encourage professionals to provide support and encourage their colleagues when they have had discussions with patients and families about recovery	Good practice guide Staff training package
	There are no standard guidelines about who should provide information about recovery, when and why. As a result, some professionals are unclear about who should provide this information.	Modelling	Demonstration of the behaviour	Be provided with examples of the roles and responsibilities of each professional and of the team in providing information about recovery	Staff role descriptions

COM-B: Capability, Opportunity, Motivation Model of Behaviour; BCT = Behaviour Change Technique. Adapted from Burton et al., 2025 [34], licensed under CC BY 4.0 (https://creativecommons.org/licenses/by/4.0/).

#### 3.3.6 Intervention implementation

Participants stressed the importance of flexibility in implementation planning, recognising variation in current practice across stroke units resulting from the lack of guidance in this area. Implementation plans therefore include a process mapping [52] session to identify current processes and opportunities to incorporate conversations about recovery across the in-patient pathway, and the staff involved who may require training. Identifying emotional support available for patients and carers was also deemed important. To monitor the impact of the intervention (including identifying any unintended consequences) and support problem-solving of challenges, regular implementation group meetings with key staff are recommended before and during intervention delivery.

## 4 Discussion and conclusion

### 4.1 Discussion

An intervention to support stroke unit staff in providing information about recovery to patients and their carers has been collaboratively developed with stroke survivors, carers and professionals. Stroke survivors and carers provided insights into how information is received and patients' preferences, whilst professionals contributed their experiences of providing information and how an intervention could be implemented in practice. The intervention emphasises a patient-centred approach, focusing on understanding the needs and preferences of patients and carers; and flexibility in implementation to enable use across stroke unit settings. Core elements include staff training and role descriptions to support skill development, good practice guidance, a tool to identify patients' needs and preferences and written information to support conversations.

At the heart of our intervention is the premise of person-centred care, supporting professionals to engage in *“personalised, coordinated and empowering”* conversations with patients and their families [53], aligned with the NHS Person-Centred Approaches framework [54]. The need for personalised information about an individual's likely recovery trajectory has been highlighted in both a recent scoping review of post-stroke information needs [55] and earlier systematic review work [15]. Our intervention advocates not just personalised information but also tailored delivery, encouraging staff to work alongside patients and carers to identify preferred types, formats and amount of information. Coordination between different members of the MDT was a key consideration in intervention design. Whilst junior staff are not expected to deliver specific predictions about recovery, participants recognised that all team members contribute to overall messaging about recovery. Supporting junior staff, e.g., healthcare assistants, in this role was considered imperative, as they are often most available to patients and carers and may frequently face questions [31]. Discussions around implementation also considered how patients and carers' information needs and preferences would be recorded, shared across the team, and regularly updated, to establish a coordinated approach. Finally, our intervention seeks to support professionals to deliver personalised information that empowers patients and carers to engage in shared decision-making and plans for discharge, including how any continuing care needs might be met.

The intervention highlights the variability in patients and carers' information needs and preferences across the stroke pathway. Whilst studies emphasise the ‘right’ information at the ‘right’ time [11], this can be challenging due to the heterogeneous effects of stroke and potential for rapid early change, with needs and preferences likely to shift over time. For example, early after stroke, patients are often acutely unwell and less able to receive information than when in rehabilitation settings [11], [55]. Carers may also experience shock at the sudden onset of stroke, limiting their ability to process information, particularly when attention is focused on immediate survival [56]. Differences in personality and culture may also have an impact. These individual differences must therefore be considered by professionals deciding when, whether and how to provide information. Our intervention advocates increased dialogue between healthcare professionals, patients and families, to better understand and respond to patients and carers' evolving needs across the pathway.

During our coproduction workshops, key themes emerged around sharing uncertain information while supporting patients to remain hopeful for their future recovery. As in previous research [57], [58], [59], stroke survivors identified hope as important for sustaining motivation during rehabilitation and coping with uncertainty. However, maintaining this balance is challenging for professionals; concerns about providing ‘false hope’ may lead to avoidance of conversations about recovery in in-patient settings. For example, Bright et al. [16] described how in-patient professionals deferred conversations about future recovery to community-based colleagues. Despite recognising fostering hope as an important part of their role [17], [60], professionals may lack confidence in achieving this balance [31]. The good practice guide and training package developed as part of our intervention offer guidance for professionals when managing these complex conversations. Their design drew upon strategies advocated in existing guidance intended to support staff in ‘breaking bad news’, e.g., Baile et al.'s SPIKES protocol [48].

### 4.2 Innovation

Given professionals' reported challenges in providing information, alongside patient and carer reports of the inadequacy of information received, conversations about post-stroke recovery have warranted increasing research attention [5], [10], [12], [16], [61], [62]. However, to our knowledge, this is the first study to address this challenge by designing an intervention to improve provision of information about recovery on stroke units. The use of ‘co’ approaches in developing interventions for post-stroke care and rehabilitation has also rapidly increased, reflecting the benefits of involving service providers and users in enhancing intervention acceptability and feasibility, and producing innovative and creative solutions to clinical problems [63]. Whilst some interventions aiming to improve information provision after stroke have been coproduced, applying these methods to improve how information about recovery is delivered is novel. This approach mirrors innovations in other conditions [27], [28] but is new in the context of stroke.

### 4.3 Strengths and limitations

Prior to this study, we had conducted substantial systematic review and qualitative work to understand processes and perspectives on providing and receiving information about recovery in stroke units, and applied behaviour change theory to identify barriers and potential ‘active ingredients’ of interventions to address them. A strength of our study is that this comprehensive work provided a strong foundation for the coproduction process. However, it may also have constrained and shaped the group's ideas and stifled creativity. For example, we had already decided to target professionals' behaviour (rather than that of patients and carers; or all three groups), based on previously identified barriers. It is possible that participants may have designed a different intervention without this direction. Despite this, participants brought their own experiences to the process and actively shaped the intervention and design of resources.

A further strength is the diversity of the stroke survivor sample in terms of age, and inclusion of professionals from the core stroke unit disciplines, including psychology. Engagement was maintained throughout the study. Collaboration between stroke survivors, carers and professionals improves the likelihood that the intervention will be acceptable and feasible to deliver in practice. However, the work was conducted in one UK city and coproduction elsewhere may have generated different results. Nonetheless, our earlier qualitative work in different locations and national stakeholder engagement may support transferability to other stroke unit contexts.

An additional limitation is the lack of ethnic diversity in our sample. Despite attempts to recruit participants from non-white backgrounds, these were unsuccessful, likely in part due to restricting recruitment to English speakers (due to resource limitations). Our previous systematic review work revealed limited research on experiences of discussing recovery in acquired neurological conditions outside of the Global North [11]. Additional work will be required to ensure our intervention is acceptable to people from diverse ethnic groups, and to support professionals working with patients and carers whose first language is not English, including consideration of family-based and formal interpretation, which may present additional challenges.

### 4.4 Conclusion

An evidence-based, theory-informed intervention has been coproduced, with the potential to meet the needs of intervention targets (staff) and information recipients (patients/carers). Further work is required to assess whether this intervention is effective in improving staff skills and confidence, and patient and carer outcomes.

## CRediT authorship contribution statement

**Louisa-Jane Burton:** Writing – original draft, Visualization, Project administration, Methodology, Investigation, Funding acquisition, Formal analysis, Conceptualization. **Thomas F. Crocker:** Writing – review & editing, Validation, Methodology, Investigation, Funding acquisition, Conceptualization. **Oliver Todd:** Writing – review & editing, Validation, Methodology, Funding acquisition, Conceptualization. **Sarah Bates:** Writing – review & editing, Validation, Resources, Project administration, Funding acquisition, Conceptualization. **Anne Forster:** Writing – review & editing, Validation, Methodology, Funding acquisition, Conceptualization.

## Ethical approval

This study received ethical approvals from the North West – Greater Manchester West Research Ethics Committee (ref: 23/NW/0243) in August 2023.

## Declaration of generative AI and AI-assisted technologies in the writing process

During the revision of this work, the authors used Microsoft CoPilot to enhance conciseness. After using this tool, the authors reviewed and edited the content as needed and take full responsibility for the content of the published article.

## Funding

This project was supported by Leeds Hospitals Charity [ref: A2002293, 2023]. Previous phases of the project (collection of qualitative data, early application of behaviour change theory, online survey) were funded by the Stroke Association through its postgraduate fellowship programme [ref: TSA PGF 2027–02].

## Declaration of competing interest

Louisa-Jane Burton reports financial support was provided by Leeds Hospitals Charity. Oliver M Todd reports financial support was provided by Leeds Hospitals Charity. Thomas Frederick Crocker reports financial support was provided by Leeds Hospitals Charity. Sarah Bates reports financial support was provided by Leeds Hospitals Charity. Anne Forster reports financial support was provided by Leeds Hospitals Charity. If there are other authors, they declare that they have no known competing financial interests or personal relationships that could have appeared to influence the work reported in this paper.

## Appendix A Supplementary data

Supplementary material 1 IMAGE 1

Supplementary material 2 IMAGE 2
