# Supplementary material for: Coproducing an intervention to support stroke unit staff to provide information about recovery to patients and carers
Source: PEC Innov. 2026 Jun 12;9:100483. doi: 10.1016/j.pecinn.2026.100483 (PMC13312195; doi:10.1016/j.pecinn.2026.100483)
Supplement: Supplementary file 1 — Supplementary material 1 [file mmc1.docx]

Supplementary File 1. Intervention description using TIDieR checklist (31).

| **Name of intervention** | inFOrmation about RECovery After STroke (FORECAST) |
| --- | --- |
| **Why: Rationale, theory, or goal for the intervention (how it is expected to work)** | Stroke unit patients and their carers desire information about recovery (including both information about the processes through which recovery takes place, e.g., neuroplasticity; and personalised predictions). FORECAST aims to improve stroke unit processes and multidisciplinary staff skills and confidence to deliver a proactive approach to information provision (primarily through verbal discussions, with supporting written information where needed), with information provided compassionately and in ways which are tailored to patients’ and carers’ individual needs and preferences. Receiving this information can be empowering and help patients and carers to plan and engage in shared decision-making about their care and promote adjustment to any on-going difficulties. The FORECAST intervention is underpinned by Behaviour Change Theory (the COM-B) and was developed using coproduction. |
| What (materials): Materials used within intervention | - Good practice guide for staff, including recommendations around the following: - Preparation for conversations about recovery; - Establishing ward processes to ensure information is proactively and routinely offered to all patients; - Discussing patients’ and carers’ expectations about recovery, their preferences for information (including whether/ how much information about recovery they want; how it should be delivered, e.g., verbal/ written, who should receive it (patient/ carer(s)) and when) and their needs (including adaptation of information according to post-stroke or other needs, including communication, cognitive, visual, English as a second language); - Tailoring information to meet patients’ and carers’ identified needs (e.g., providing written information to support conversations); - Delivery of information (clarity, honesty, kindness; supporting patients’ and carers’ hope; managing uncertainty; breaking bad news); - Maintaining consistency in messaging across the MDT; - Developing patients’ and carers’ understanding of the role of rehabilitation and services in post-stroke recovery. - Descriptions of roles of different team members of varying experience levels, with expected skills (Staff Role Descriptions). - Structured checklist to support discussion with patients and carers about their expectations, information preferences and needs (Communication Starter Tool). - Visual map of the patient journey (flexibility to tailor to each stroke unit using information from process mapping), including information about existing services, how provision acts to support the recovery process at each stage of the patient’s journey and length of stay/ treatment times (Patient Journey Map). |
| What (procedures): Procedures, activities, and processes | - Process mapping meeting to identify current process in providing information about recovery and staff involved (including available emotional support); to facilitate tailoring of intervention materials (e.g., patient journey map) and develop localised implementation plans. - Staff participation in interactive training programme, including Powerpoint presentations; opportunities for questions; guided group discussion; demonstration of communication skills; role play with feedback; and reflection. - Staff implement process to ensure routine offer of information to each patient; engage in collaborative discussion to identify patients’ and carers’ information preferences and needs; provide patient journey map; provide both generic and personalised information about recovery to patients and carers in line with good practice guidelines (primarily through discussion, supported with written materials where needed). - Regular implementation groups for staff to help problem-solve during intervention delivery. |
| Who provided: Expertise, training given | Staff training provided by researcher and a palliative care nurse with experience in training healthcare professionals in communication skills and breaking bad news. |
| How: Mode of delivery | Individual receipt of good practice guide. Face-to-face group-based programme (groups of ~10 multidisciplinary staff of all experience levels) with some individual work. |
| Where: Locations where intervention was delivered | Local training rooms at hospital site. |
| When and how much: Timing and intensity | 1 x 3.5 hour session (all staff) plus 1 x 3.5 hour additional session for qualified staff involved in providing complex information about recovery, including breaking bad news. Advance provision of good practice guide. |
| Tailoring: Planned personalisation of the intervention | Implementation plans tailored for each site through process mapping, e.g., identifying staff requiring training, and implementation groups for problem-solving.  Patient journey map adapted for each site to reflect service provision. |
| How well (planned): If/ how fidelity was assessed | Regular implementation groups for staff to help problem-solve during intervention delivery. |

COM-B = Capability, Opportunity, Motivation model of Behaviour; MDT = Multidisciplinary Team
