# Supplementary material for: Coproducing an intervention to support stroke unit staff to provide information about recovery to patients and carers
Source: PEC Innov. 2026 Jun 12;9:100483. doi: 10.1016/j.pecinn.2026.100483 (PMC13312195; doi:10.1016/j.pecinn.2026.100483)
Supplement: Supplementary file 2 — Supplementary material 2 [file mmc2.docx]

Supplementary File 2. Completed GUIDED checklist

GUIDED – a guideline for reporting for intervention development studies (35)

|  | Item description | Page in manuscript where item is located |
| --- | --- | --- |
| 1 | Report the context for which the intervention was developed. | 4-5, 10-12 |
| 2 | Report the purpose of the intervention development process. | 7, 8 |
| 3 | Report the target population for the intervention development process. | 7 |
| 4 | Report how any published intervention development approach contributed to the development process | 7-8 |
| 5 | Report how evidence from different sources informed the intervention development process. | 7-8 |
| 6 | Report how/if published theory informed the intervention development process. | 7-9, 21 |
| 7 | Report any use of components from an existing intervention in the current intervention development process. | N/A |
| 8 | Report any guiding principles, people or factors that were prioritised when making decisions during the intervention development process. | 8 |
| 9 | Report how stakeholders contributed to the intervention development process. | 14-21 |
| 10 | Report how the intervention changed in content and format from the start of the intervention development process | 14-20 |
| 11 | Report any changes to interventions required or likely to be required for subgroups. | 37 |
| 12 | Report important uncertainties at the end of the intervention development process. | 37 |
| 13 | Follow TIDieR guidance when describing the developed intervention. | Supplementary File 1 |
| 14 | Report the intervention development process in an open access format. | Intention to publish in OA format |
